# Au-Nanorods Supporting Pd and Pt Nanocatalysts for the Hydrogen Evolution Reaction: Pd Is Revealed to Be a Better Catalyst than Pt

**DOI:** 10.3390/nano13132007

**Published:** 2023-07-05

**Authors:** Ayoub Laghrissi, Mohammed Es-Souni

**Affiliations:** 1 Currently with the Technical Faculty, Mads Clausen Institute, University of Southern Denmark, 6400 Sonderborg, Denmark; laghrissi@mci.sdu.dk; 2 Formerly with Kiel University of Applied Sciences, Grenzstrasse 3, D-24149 Kiel, Germany

**Keywords:** hydrogen evolution reaction, bimetallic Au-Pt-Nanorods, bimetallic Au-Pd-Nanorods, nanoelectrocatalysts, hydrogen adsorption energy, density functional theory

## Abstract

Ordered thin films of Au nanorods (NRs) on Ti/Au/Si heterostructure substrates are electrodeposited in thin film aluminum oxide templates and, after template removal, serve as supports for Pd and Pt nanocatalysts. Based on previous work which showed a better electrocatalytic performance for layered Au/Pd nanostructures than monolithic Pd, electrodeposited 20 nm Pd discs on Au-NRs are first investigated in terms of their catalytic activity for the hydrogen evolution reaction (HER) and compared to monolithic 20 nm Pd and Pt discs. To further boost performance, the interfacial interaction area between the Au-NRs supports and the active metals (Pt and Pd) was increased via magnetron sputtering an extremely thin layer of Pt and Pd (20 nm overall sputtered thickness) on the Au-NRs after template removal. In this way, the whole NR surface (top and lateral) was covered with Pt and Pd nanoparticles, ensuring a maximum interfacial contact between the support and the active metal. The HER performance obtained was substantially higher than that of the other nanostructures. A Salient result of the present work, however, is the superior activity obtained for sputtered Pd on Au in comparison to that of sputtered Pt on Au. The results also show that increasing the Au-NR length translates in a strong increase in performance. Density functional theory calculations show that the interfacial electronic interactions between Au and Pd lead to suitable values of hydrogen adsorption energy on all possible sites, thus promoting faster (barrier-free diffusion) hydrogen adsorption and its recombination to H_2_. A Volmer–Heyrovsky mechanism for HER is proposed, and a volcano plot is suggested based on the results of the Tafel plots and the calculated hydrogen adsorption energies.

## 1. Introduction

The ever-increasing share of renewables in the energy mix is understandably driven by environmental concerns. In many regions of the world, the share of renewables is now at least 20% and is expected to increase during the coming decade to 40 or even 50% [1,2]. Primarily, this trend has been boosted by recent developments in Li-ion battery modules that have become much more competitive as Li’s price in the world market continues to fall [3]. Nevertheless, the efficient exploitation of renewables also involves other energy storage possibilities, such as (super)capacitors and water electrolysis (to mention only the electrochemical methods). In particular, water electrolysis, mainly for the production of hydrogen via the hydrogen evolution reaction (HER), is highly attractive, could provide a powerful means of achieving clean and sustainable energy storage that could be used even in remote and desertic regions.

HER is a complex electrochemical interfacial process that follows specific mechanisms depending on pH, catalyst, and applied voltage. These mechanisms and the type of reactions involved at the interface between electrolyte and catalyst were outlined in a review article by Conway and Tilak [4]. The generally accepted reaction steps using a metal (M) catalyst under acidic conditions are as follows [4,5,6]:

(1) $$H_{3}O^{+}+M+e^{-}\to {MH}_{ads}+H_{2}O\;\;(\mathrm{Volmer}\;\mathrm{reaction})$$

followed by either reaction (2) or (3)

(2) $${MH}_{ads}+H_{3}O^{+}+e^{-}\to H_{2}↑+{M+H}_{2}O\;\;(\mathrm{Heyrovsky}\;\mathrm{reaction})$$

(3) $${2MH}_{ads}\to H_{2}↑+2M\;\;(\mathrm{Tafel}\;\mathrm{reaction})$$

The mechanisms of HER have been rationalized by different authors in terms of the governing equations specific to the mechanisms above, taking into account the different acting kinetics parameters such as adsorbed hydrogen surface coverage and overpotential (e.g., Conway and Tilak [4], Bhardwaj et al. [7]). A number of catalysts have been reported for the HER, including transition metals and various compounds, as compiled in the review article of Eftikhari [8]. Catalyst performance is generally weighed in terms of the magnitude of overpotential necessary for HER at a given pH, the current density generated/weight of the active catalyst, and durability. The noble metals (Pt, Pd) that are mostly used in acidic conditions are well known for their outstanding performance [9,10,11,12]. In particular, Pt-NPs, to date, are considered the best known HER catalysts, performing at very low overpotentials. Although other non-noble metal catalysts such as Ni and Ni-Mo [13], as well as non-metallic catalysts, including FeS_2_, MoS_2_ [14], and C_3_N_4_ [15], are now in the focus of research, Pt-group metals still attract interest, particularly with respect to strategies to decrease their loading and the governing HER mechanisms (see results and discussion below).

In a previous work, we showed that layered Au-Pd-nanorods (NRs) are powerful electrocatalysts for formic acid electrooxidation [16]. In this prior study, controlling the Pd-layer thickness to few nanometers was shown to drastically increase the performance of the catalyst. Further, by means of functional density calculations, it was shown that Au plays an active role in preventing catalyst poisoning by catalyzing the oxidation of CO. Based on this work, the present study explores the feasibility of these catalysts for HER. In particular, it is shown that increasing the interfacial contact area between Au and the active metals Pd and Pt results in performances that vastly surpass those of the layered and monolithic Pt and Pd nanostructures. This is achieved by magnetron sputtering a very thin layer of Pt and Pd (overall 20 nm thickness of the metals) on exposed Au-NRs (i.e., NRs after removal of porous aluminum oxide template), which leads to nanoparticle formation and distribution on the top and lateral surfaces of the Au-NRs, thus maximizing the interfacial contact area between the support and the active metal. Our results unambiguously show that sputtered Pd on Au-NRs yields a substantially higher HER performance than that of sputtered Pt on the same Au-NRs. The results are rationalized in terms of hydrogen adsorption energy on different sites of Au-Pd(Pt)-bimetallic structures using density functional theory calculations.

## 2. Materials and Methods

### 2.1. Synthesis and Characterization

The following chemicals were used as purchased: oxalic acid dihydrate 99% (Roth, Karlsruhe, Germany), phosphoric acid 85% (Roth, Karlsruhe, Germany), and sodium hydroxide (Roth, Karlsruhe, Germany); Gold (III) Chloride Trihydrate (Sigma-Aldrich, Steinheim, Germany); Potassium tetrachloropalladate(II) (abcr, Karlsruhe, Germany); and Chloroplatinic acid hexahydrate (Sigma-Aldrich, Steinheim, Germany). Deionized water was used to prepare aqueous solutions.

Aluminum thin films of approximately 500 nm were deposited on a Silicon/Ti (2 nm)/Au (5 nm) heterostructure via electron beam evaporation (PVD75, Lesker, Jefferson Hills, PA, USA) equipped with an electron beam system (Ferrotec GmbH, Unterensingen, Germany). Anodization was conducted potentiostatically in 0.1 M oxalic acid at 70 V in a two-electrode set-up with a Pt mesh as the counter electrode, using an electrochemical workstation (Keithley 2400 SM, Cleveland, OH, USA). After anodization, barrier layer removal, and pore widening were conducted in phosphoric acid (5 wt.% in water) for 50 min at 30 °C [17,18]. Au-NRs, Pt-NRs, and Pd-NRs were grown into the AAO pores via electrodeposition in 8 mM HAuCl_4_, 10 mM H_2_PtCl_6_, and 10 mM K_2_PdCl_4_ aqueous electrolyte using a three-electrode set-up with the Au/AAO template working electrode (WE), a Pt counter electrode (CE), and an Ag/AgCl reference electrode under potentiostatic conditions (Au at 0.1 V, Pt at −0.2 V, and Pd was first initiated at −0.1 V for 30 s and subsequently the voltage was increased to 0.3 V) using an electrochemical workstation (Princeton Potentiostat/Galvanostat Model 263A, Oak Ridge, TN, USA). Au/Pd-NRs were grown into the pores via sequential electrodeposition from aqueous electrolytes. The NRs length was controlled via the deposition time. After electrodeposition, the 1D nanostructures were exposed by dissolving the AAO film in an aqueous solution of NaOH (5 wt.%) at room temperature.

Subsequently, the supported Au-NRs were introduced into the sputtering chamber. Next, 20 nm thick Pt and Pd films were RF sputtered on the Au-NRs samples and the supporting substrate using pure targets (Pt and Pd (ESG Edelmetall GmbH, Rheinstetten, Germany)) under an approximate argon partial pressure of 10-3 mbar. The sputtering power was set to 100 W for Pt and 120 W for Pd (at room temperature). The film thickness was monitored by using a quartz crystal microbalance. The sputtering of the 20 nm Pt layer on a Silicon/Ti (2 nm)/Au substrate (the same substrate used to grow the Au-NRs) was introduced together with Au-NRs to the sputtering chamber.

The microstructure and morphology of the samples were characterized by using a high-resolution scanning electron microscope (SEM Ultra Plus, ZEISS, Oberkochen, Germany) operating in the secondary (SE) and energy-selective backscattered (ESB) electron modes. The SEM is also equipped with an energy-dispersive X-ray spectroscopy (EDS) package (INCAx-act, Oxford Instruments, High Wycombe, UK). The structure was characterized by X-ray diffraction (XRD, X’Pert Pro diffractometer PANalytical, Eindhoven, The Netherland) in grazing incidence diffraction mode with constant θ = 1° using monochromatic CuKα radiation with λ = 1.5418 Å. The device has a full width at half maximum resolution of 0.03°. 

An electrochemical workstation (ZAHNER IM6e, Kronach, Germany) was used for linear sweep voltammetry (LSV) measurements from −0.8 V to 0.3 V. The electrochemical experiments were performed in 0.5 M H_2_SO_4_ solution with pH 0.36 using a three-electrode set-up with a Pt mesh and HydroFlex (reversible H_2_ reference electrode) as counter and reference electrodes, respectively. All potentials were referenced to the reversible hydrogen electrode (RHE). The current was normalized by the sample’s measured area. All the H_2_SO_4_ solutions were saturated with Forming gas N_2_+H_2_ (after the usual bubbling with nitrogen) before measurement. All the data presented were corrected for iR losses and background current. Electrochemical impedance spectroscopy (EIS) measurements were performed at frequencies between 0.1 and 100,000 Hz. The EIS data were fitted to determine the solution resistance, charge transfer resistance, and capacitance using Randle’s circuits (Figure S1).

### 2.2. Computational Methods

Ab initio calculations based on density functional theory implemented in Quantum ESPRESSO v.6.0 package were employed to simulate the adsorption of hydrogen on different (111)-oriented Au, Pt, Pd, AuPt, and AuPd sites [19]. The electron–ion interaction was described via projector augmented wave (PAW) potentials, while the exchange correlation was represented via generalized gradient approximation (GGA) using the Perdew–Burke–Ernzerhof (PBE) framework [20]. The plane wave basis cut-off energy was set to 400 eV. The structures were initially optimized by permitting the relaxation of atom positions by using the Broyden–Fletcher–Goldfarb–Shanno (BFGS) method. To achieve high accuracy, the self-convergence-field convergence criterion was set to 10^−6^ eV. 

**Figure 1:**
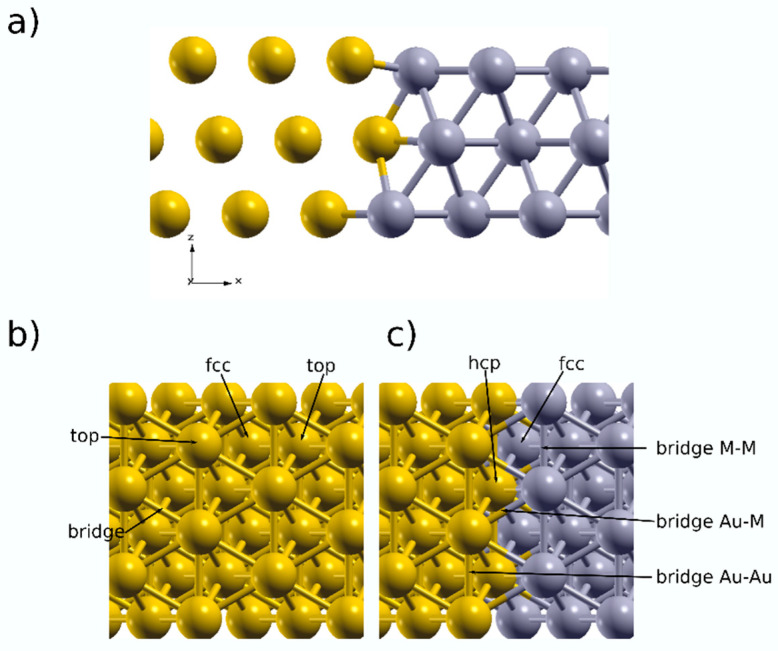
(**a**) side-view of the AuPd and AuPt slab, with possible adsorption sites for: (**b**) Au, Pt, and Pd (111), (**c**) AuPd, AuPt (111).

Furthermore, (111)-oriented Au, Pt, Pd, AuPt, and AuPd surfaces were modelled with the 4 × 3 × 2 slab. The AuPt (AuPd) were modeled by placing two slabs of Au and Pt(Pd) side by side along the *x*-axis (striped structure) (Figure 1a), then the atomic layer was left relaxed. This particular geometry was chosen to study the adsorption of hydrogen at the interface between Au and M on the 111-surface. Figure 1b shows different hydrogen adsorption sites, namely, top, bridge, fcc, and hcp for Au, Pt, and Pd. For AuPt and AuPd, hydrogen adsorption was examined on the Au/Pt (Au/Pd) interface; in this case, the new possible adsorption sites were as follows (Figure 1c): bridge sites Au–Au and Pt–Pt (Pd–Pd). The hydrogen atom and the top layer of the slab were allowed to relax their atomic positions to attain the most stable surface–hydrogen distance (dS–H). Hydrogen adsorption energies were calculated on the basis of the usual definition of Equation (4):

(4) $$E_{ads}=E_{Metal–H}-E_{H}-E_{Metal}$$

## 3. Results

### 3.1. Structure and Morphology 

**Figure 2:**
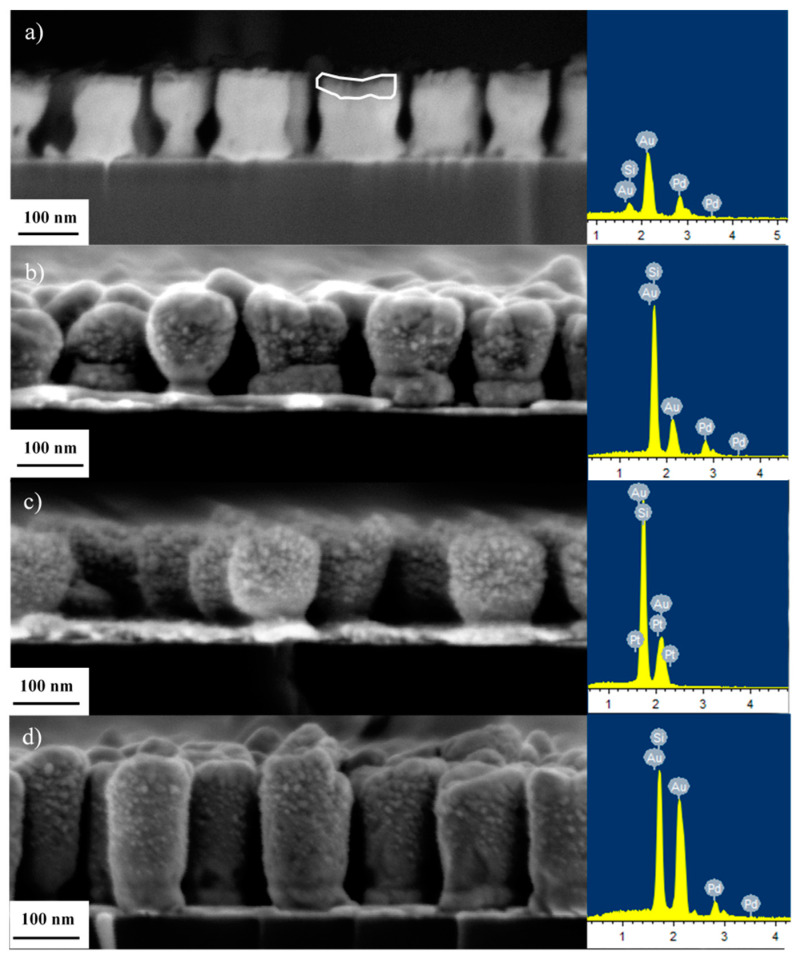
SEM micrographs and EDS spectra of (**a**) layered Au/Pd-NRs, (**b**) Au-NRs/Pd (sputtered) 100/20 nm, (**c**) Au-NRs/Pt (sputtered) 100/20 nm, (**d**) Au-NRs/Pd (sputtered) 200/20 nm; (**a**) shows a high-resolution back-scatter electron micrograph of the electrodeposited Pd-caps (delineated), approximately 20 nm thick on top of the Au-NRs; sputtering of Pd (**b**,**d**), and Pt (**c**) on the Au-NRs leads to complete coverage of the Au-NRs surface area with Pd and Pt-NPs.

Here, we focus on the Au/Pd(Pt) nanostructures. The structure and morphology of the monolithic nanostructures are shown in the Supplementary Materials (Figure S2). A high-resolution back-scatter electron micrograph of electrodeposited Au/Pd 100/20 nm NRs is shown in Figure 2a, together with the corresponding EDS spectrum. Based on atomic number contrast, Pd (outlined, dark-contrast layer) forms caps of mean thickness of 20 nm on the Au-NR tips. There is no evidence to suggest that Pd is deposited on the lateral surface of the NRs, e.g., compare Figure 2a with Figure S2a in the Supplementary Materials, where smooth lateral surfaces can be seen in both cases. In another experiment and in order to explore the effect of the deposition method and Pd (Pt) morphology, 20 nm thick layers of Pd and Pt were also deposited on the Au-NRs (after template removal) via physical vapor deposition from pure Pd and Pt targets. In this case, Pd and Pt are deposited on the whole surface area of the Au-NRs. By comparing Figure 2b–d to supplementary material Figure S2a, it is clear from observing the rough morphology and the particulate structure of the NR surfaces that Pd and Pt distribute as NPs over the whole surface of the NRs. 

**Figure 3:**
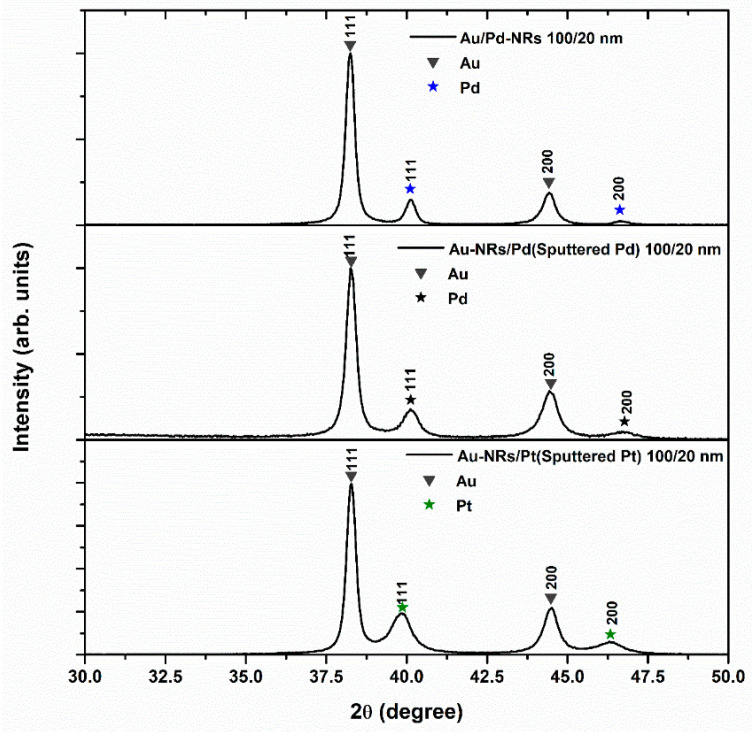
XRD patterns in the range of 111 and 200 reflections in grazing incidence mode.

The XRD patterns of the nanostructures investigated in this study are shown in Figure 3 with the usual reflections of Au, Pd, and Pt. In the case of sputtered Pd and Pt, a broadening of the peaks can be observed, underscoring the nanoscale dimensions of the deposited materials. The mean particle size calculated using the Scherrer formula is 26 nm for electrodeposited Pd, 15 nm for sputtered Pd, and 12 nm for sputtered Pt. The ratios of the 200 to 111 peak areas are 0.19 for Pd and 0.37 for Pt, delineating the predominance of the 111 orientation in both cases. 

### 3.2. Electrochemical Investigations, HER

**Figure 4:**
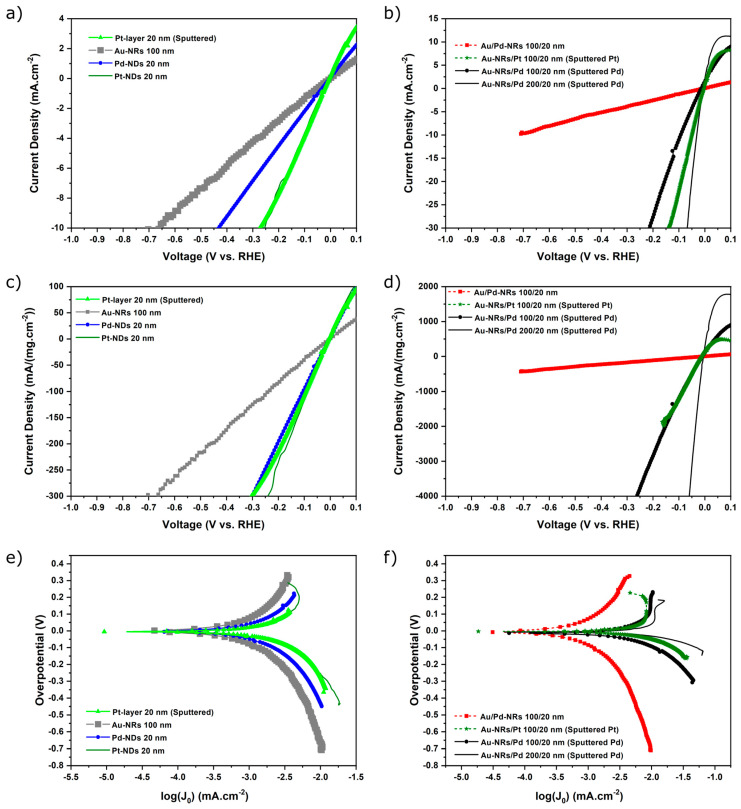
IR-corrected linear sweep voltammograms in 0.5 M H_2_SO_4_ of (**a**,**c**) monolithic and (**b**,**d**) bilayer structures with electrodeposited Pt and Pd as well as sputtered Pt and Pd on Au-NRs. The current density is normalized by the geometric surface area in (**a**,**b**) and by catalyst loading in (**c**,**d**). Current density values at overpotentials of −0.1 V are listed in Table 1 for the different catalyst nanostructures. (**e**,**f**) Tafel plots corresponding to the LSV curves. Sputtered Pd and Pt nanostructures are indicated. All others were electrodeposited. The current density in the Tafel plots refers to the geometric surface area. The Tafel slopes are listed in Table 1.

The linear sweep voltammetry (LSV) results, as well as corresponding Tafel plots in 0.5 M H_2_SO_4_ electrolyte (pH = 0.36), are displayed in Figure 4. The results clearly reflect that the electrocatalytic performance very much depends on the processing method of the catalyst. 

Interestingly, the Au-NRs show a catalytic activity of approximately −1.24 mA/cm^2^ difference at −0.1 V. Few reports have documented that Au-nanostructures show HER activity. For instance, Kiani et al. reported the moderate catalytic activity of nanoporous Au-films [21], though at fairly higher overpotentials as the ones observed in this work, and similar results to ours were obtained on Au-aerogel supported on graphitic carbon nitride and Au-NPs by Kundu et al. [22].

The electrodeposited layered and monolithic nanostructures, including Pt, which is known to be the best catalyst under acidic conditions, all show moderate performance in the range from approximately −4 (for sputtered 20 nm Pt layer) to −1 mA/cm^2^ at −0.1 V (see Table 1). It can also be seen that Au barely affects the performance of electrodeposited Pd. 

A performance increase is obtained with sputtered Pt on Au-NRs in comparison to the sputtered Pt film, which is understandable due to the NP-morphology and the increase in the surface area of Pt. However, sputtered Pd on Au-NRs yields a substantially higher HER activity, that is, in terms of current density (at a constant overpotential), more than seven times that of comparable, electrodeposited Pd nanostructures, e.g., at −0.1 V (see Table 1). Using 200 nm Au-NRs supports further improves the performance, which is amenable to the higher surface area of Pd-NPs achieved with longer Au-NRs. Comparing sputtered Pt and Pd on the 100 nm long Au-NRs, it can be seen that they have matching performances in terms of gravimetric current density (Figure 4d). However, increasing the interfacial area between Pd and Au-NRs by using 200 nm long Au-NRs results in a substantial increase in the HER performance, beyond that of Pt/Au-NRs (100/20 nm). Because it is known that Pd-NPs on carbon (Pd/C) have a substantially lower activity than Pt/C [23], it appears that combining Pd-NPs with Au in an Au/Pd bimetallic nanostructure improves the catalytic activity. Feng et al. [24] also reported that AuPd bimetallic nanocrystals are more efficient a catalyst than Pd/C, although their results show that Pt/C is a slightly better catalyst. From our results, we can surmise that the nature of electronic interactions at the Pd (Pt)/Au interface must play a key role in controlling catalyst performance (see below for discussion). 

The Tafel plots are displayed in Figure 4e,f. For all structures, at least two different slopes can be distinguished; at overpotentials higher (in absolute value) than −0.1 V (versus RHE), the current density exponentially depends on the overpotential. This behavior has been reported by Conway and Bai [25] and was rationalized in terms of the hydrogen surface coverage’s dependence on the overpotential using the Volmer–Heyrovsky mechanisms [25,26].

The slopes calculated at low overpotentials (in absolute value) are listed in Table 1. They range from values of 139 to 24 mV/dec, probably reflecting different structure-dependent HER mechanisms (see below for discussion). The Au-NRs show the highest slope (139 mV/dec), followed by the slopes of the electrodeposited Pd (96 mV/dec) and layered Au/Pd (94 mV/dec) nanostructures. The electrodeposited Pt-nanodiscs (53 mV/dec) and sputtered Pt layer (57 mV/dec) show intermediate values that are higher than what is known for carbon supported Pt-NPs [27] but lower than the values reported for polycrystalline Pt [28,29]. For the Au/sputtered Pt and Pd nanostructures, the values decrease to roughly 24 mV/dec for Au/Pt and 37 mV/dec for Au/Pd, although the lowest value is shown by Pd sputtered on the longer 200 nm Au-NRs. 

From the Tafel slopes above, we can definitively state that the operating HER mechanisms are dependent on structure and the morphology of the material. Particularly, the Au/sputtered Pd nanostructures seem to stand out due to the fact that they yielded both the highest performance and lowest Tafel slope. 

**Figure 5:**
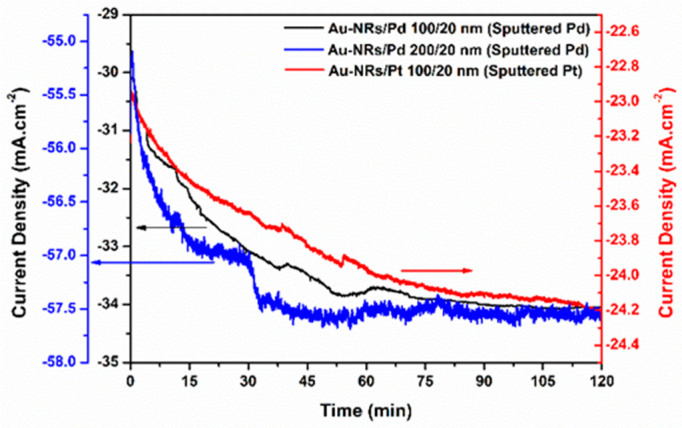
Chronoamperometric curves of Au-NRs/Pt 100/20 nm, Au-NRs/Pd 100/20 nm, and Au-NRs/Pd 200/20 nm in 0.5 M H_2_SO_4_ at −0.4 V for 120 min.

Chronoamperometric measurements were conducted in 0.5 M H_2_SO_4_ at a fixed voltage of −0.4 V for 120 min to test the long-term property stability and the aging of structures under critical conditions, where the system is subjected to higher stress levels via a more negative potential, similar to previous works [30] (Figure 5). It can be seen that the activity of both Au-NRs/Pd and Au-NRs/Pt continually increase for some time before reaching saturation, probably because of the activation and full coverage of all surface sites. As expected, Au-NRs/Pd saturates at a higher value (absolute value) than Au-NRs/Pt. Further, Au-NRs/Pd saturates faster than Au-NRs/Pt, which might be attributed to their fast diffusion of and coverage with hydrogen because of lower potential barriers. The large fluctuation in the chronoamperometry curves can be explained by a sequence of adhesion of the hydrogen bubbles to the electrode surface followed by diffusion and floating of the bubbles, proving that the hydrogen bubbles do not highly obstruct and block the electrode surface. We should now shed some light on these observations via DFT calculations, particularly considering the effect of hydrogen adsorption energies on different sites of the structures. 

### 3.3. DFT Calculations

Table 2 summarizes the calculated H adsorption energies on different sites of monolithic, (111)-oriented Au, Pt, and Pd surfaces. Obviously, the hydrogen adsorption energy is material- and site-dependent, with Au (111) being characterized on all sites by the lowest hydrogen adsorption energies. Pt and Pd exhibit competitive site stability, with the strongest hydrogen adsorption on fcc Pd(111). The results obtained are comparable with those reported by Ferrin et al. [31], with a difference of 10~40 meV, due to the different types of functional used. Ferrin et al. used GGA-PW91, while in the present work, GGA-PBE functional was used, which tends to over-bind hydrogen to the metals. Mattson et al. [32] examined the differences between GGA-PW91 and GGA-PBE functionals, finding that both could lead to much larger differences than those obtained using different DFT codes. As different events occur in the HER process, including hydrogen adsorption, hydrogen diffusion, and H_2_ formation, the low stability of Au sites for hydrogen adsorption might point to the discharge of hydronium ions on Au as the rate-limiting step (Volmer reaction), which also explains the high Tafel slope obtained. By comparing the adsorption energy on the different sites, it can be seen that the Pt sites are characterized by closer values (variance of 0.11 eV) than those of Pd (variance of 0.63 eV). Since the capacity of hydrogen diffusion over the catalyst surface facilitates easy surface coverage with hydrogen as well as the recombination of the adsorbed hydrogen according to the diffusion mechanisms explained by Watson et al. [33], the hydrogen diffusion on the catalyst surface involves hopping from one site to another until full coverage of the surface is achieved. When the adsorption energy at one site is much larger, that site becomes a barrier for hydrogen to pass through to the next site, making the diffusion process difficult and, consequently, also full coverage of the catalyst surface difficult as well; thus, the diffusion of hydrogen over the surface is progressively more rapid on Pt than over Pd. In the case of Pd, the diffusion pathway is through the top and bridge site; hence, compared to Pt, diffusion over the Pd surface is hampered, as H adsorbates need to go through the top site, leading to a critical energy barrier. 

Bimetallic nanocatalysts behave differently, as depicted in the experimental results above. To understand these behaviors, two (111) layers of Au and M (Pt, Pd) were placed in lateral contact (Figure 1c) and left for the relaxation of atomic positions.

Additional adsorption sites arise from this procedure: two top Au and M (Pt, Pd) sites, three bridge Au–Au, Au–M, and M–M sites, and several hollow sites. Since the sputtered layer covers the whole Au-NR surface (leading to a core–shell like structure), the relevant sites for H-adsorption should be Au m and M-M. Table 3 summarizes the adsorption energies and the distance from the surface for different sites of (111)-oriented AuPd and AuPt surfaces. The first observation is that the top Au and bridge Au–Au become more stable for both surfaces compared to the bare Au surface (difference of >0.6 eV), but this is rather secondary to our context, since Au is buried under the Pt/Pd metal layer. Compared to bare Pt and Pd, the top sites become the most favorable sites for hydrogen adsorption. Comparing the present results to others, Hu et al. also similarly reported that the top-site adsorption of hydrogen on platinum–gold nanoparticles are more stable for all Pt contents of AuPt–NPs than the top site of pure Pt–NPs [34]. The variance in adsorption energies between different sites is 0.26 eV for AuPd and 0.78 eV for AuPt. The hydrogen diffusion ability over the surfaces becomes easier for the Pd-containing surface than for the Pt-containing one, leading to a high hydrogen coverage of the surface for AuPd and helping to boost the HER activity, as shown in the experimental results. 

**Figure 6:**
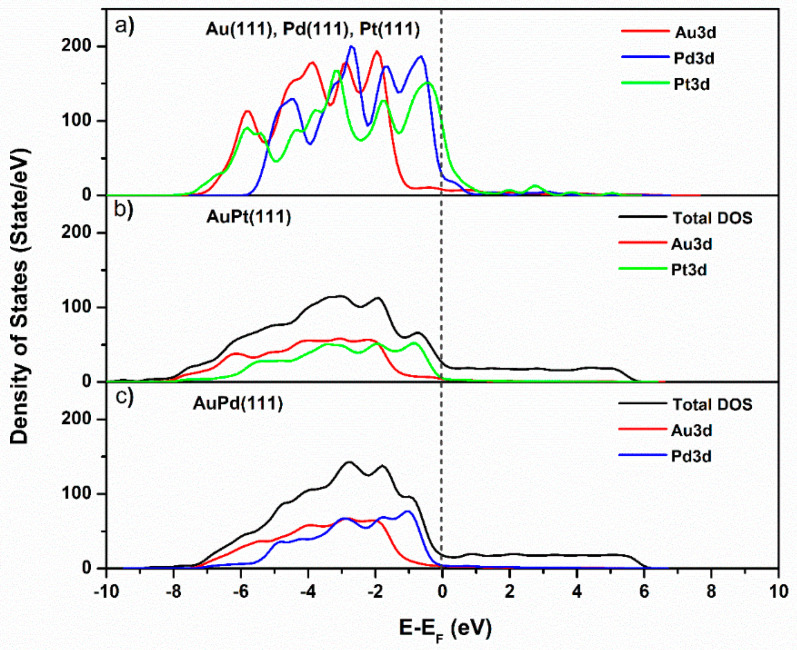
Orbital electronic density of states for 111-oriented (**a**) Au, Pt, Pd, (**b**) AuPt, (**c**) AuPd. E_F_ denotes Fermi level. Notice the change in the occupied states distribution and intensities for Pt in AuPt and Pd in AuPd.

By considering the density of states, the different behaviors of AuPd and AuPt may be understood. Figure 6 shows the density of states calculated for the pure metals and the AuPt and AuPd surfaces. The results are in qualitative agreement with the results published so far on Au, Pt, and Pd for pure metals [35,36,37]. The d-states occupation of the metals in AuPt and AuPd largely differs from those of the pure metals, both in intensity and energy range (compare Figure 6a to Figure 6b,c).

The reactivity of bimetallic catalysts is usually discussed in terms of ligand and strain effects. In the present work, the system is assumed to be mechanically relaxed, i.e., the strain effects are neglected and the ligand effects are the main focus. While this might not reflect the true situation at the interface, the present DFT could provide an additional rationalization tool for the experimental results. 

The interaction between hydrogen and the metal is directly proportional to the distance between the d-band center and the Fermi level of the metal [38,39,40]. The interaction energy can be described by Equation (5) [38,39,40]:

(5) $$∆\varepsilon \sim \frac{V²}{\left|\varepsilon_{d}-\varepsilon_{H}\right|}$$

where V is the coupling matrix (assumed to be constant for similar configurations); |ε_d_ − ε_H_| is the d-band shift, which controls the interaction energy and consequently the bond formation. In the monolithic metals, the calculated d-band centers are −2.82 eV, −2.33 eV, and −2.62 for the Pt, Pd, and Au surfaces, respectively. In the case of the AuPt and AuPd interfaces, the d-band center gradually shifts to higher energies for Pt and lower energies for Pd, toward the Fermi level (−2.77 eV for Pt, −2.96 eV for Au in AuPt, −2.51 eV for Pd, and −2.58 eV for Au in AuPd). When Au is alloyed with Pt, the charges are directly transferred from the Pt d-band to fill the empty states of the Au d-band [36], leading to a shift in the d-band center to lower energies for Au and higher energies for Pt. This result is in agreement with those reported by Mott et al. [35], who also observed a linear Pt d-band center shift with increasing Au concentration in AuPt-NPs. 

**Figure 7:**
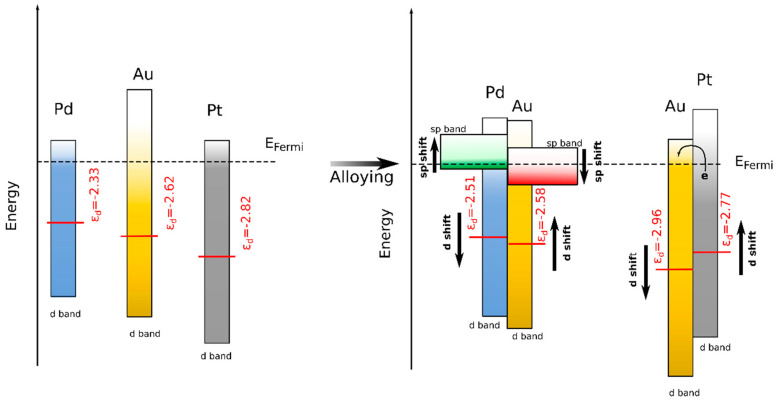
Schematic representation of the d-band shift in Au, Pt, Pd, AuPt, and AuPd. ε_d_ is the d-band centre. A direct charge transfer from Pt to Au results in a d-band shift to higher energies for Pt in AuPt; the charge transfer mechanisms in AuPd are more complex and involve the sp-band as a mediator for the charge redistribution in Au and Pd.

In the case of AuPd, Lee et al. investigated the redistribution of charges in an AuPd alloy [37]. They concluded that Au loses d-charges and gains sp charges together with the depletion of Pd sp charges and the gain of d charges. This charge redistribution leads to a small net charge transfer from Pd to Au. In this case, there is a transfer of charges from Pd to Au, but this is accompanied with sp-d hybridization, which compensates the charges by injecting them into the Au sp band. This explains the charges added into the Pd d-band (d-band center shift) with the preservation of the charge transfer predicted by the difference in electronegativity (see Figure 7 for a schematic description of the d-band shift).

Combining the calculated adsorption energies with the d-band center model, the introduction of Au into the matrix should moderately favor the adsorption of H on Pt (|ε_d_ − ε_H_| decreases) while slightly decreasing adsorption affinity on Pd (|ε_d_ − ε_H_| increases). In all cases, however, full coverage of the metal surface (Pt, Pd) with hydrogen is expected, but AuPd should be a better catalyst owing to the lower hydrogen adsorption energy and the lower d-band center shift. This result is principally in line with the results of Kibler et al. [41], who investigated pseudomorphic Pd monolayers on Au (111) and explained their findings from the point of view of ligand theory (a change in the interatomic distance of the Pd monolayer, which consequently alters the d-band center [40]). 

## 4. Discussion

The experimental results depicted above reveal that while the monolithic nanostructures all show a moderate HER activity, including a sputtered Pt-layer and electrodeposited Pt-nanodiscs, a drastic increase in performance occurs when thin layers of Pt and Pd are sputtered on Au-NRs, allowing for a large interfacial area between the active metal and the support to be achieved. Thus, the structure obtained is akin to a Au-core-Pt (Pd)-shell. Additionally, It has been extensively discussed that the phenomenon of miscibility in metals is crucial in determining the electronic interactions and the general properties of materials [42,43,44]. As a result of the complete miscibility observed in the Pd-Au system, substituted solids with enhanced electronic properties can be formed. On the other hand, Pt-Au presents an interesting case due to its large miscibility gap, which leads to phase separation and a difference in electronic properties. Regarding the present work, it is thought that the main interactions between the metals are solely at the interfacial level since no thermal treatment that could have caused intermixing was performed after sputtering. This unambiguously suggests that interfacial electronic interactions in bimetallic Au/Pt and Au/Pd are of paramount importance in controlling the electrocatalytic activity. Comparing the bimetallic Au-NRs/sputtered Pd and Pt catalysts, it is clear that Au/Pd is far more active than Au/Pt, which must be amenable to specific changes in the density of state of Pd and d-band center shift, as discussed above, endowing it with higher performance. This finding is particularly interesting since Pt (e.g., Pt/carbon) is known to be a better catalyst than Pd (e.g., Pd/C) [23,24,41].

Recently, it has been argued that when Pt is used as the counter electrode (CE) in HER studies, Pt may dissolve at high positive voltages and deposit on the working electrode (WE) [45]. It has been shown that the extent of dissolution primarily depends on the lower potential limit, which determines the magnitude of the positive voltage on the CE. However, these studies were conducted on carbon cloth WE and MoS_2_ NPs on glassy carbon. Further, the maximum CE area used was the same as that of the WE. The choice of carbon cloth as WE may also explain the chemical dissolution of Pt observed by other authors.

Tian et al. investigated the influence of the ratio of the geometric surface area of WE to CE (both of Pt) on the dissolution of Pt in 0.5 M H_2_SO_4_ upon potential cycling [46]. The main result of their work may be summarized in the following terms: when WE/CE is higher than 1, Pt dissolution from CE takes place. Pt dissolution is further magnified when O_2_ is dissolved in the electrolyte. However, Pt dissolution from CE is negligible (under the detection limit of plasma coupled mass spectrometry) when WE/CE is 1:2 or 1:10, and the electrolyte is purged of O_2_. This situation applies to the present work. We compared our working and counter electrode area ratios (CE geometric area is 4.8 cm^2^, WE geometric area is 1.326 cm^2^, ratio of 0.276; the electrolyte was also saturated with H_2_/N_2_) with their results. The maximum dissolved Pt mass should not exceed 200 ng/cm^2^, which is less than 1 surface monolayer of Pt. Further, after the HER measurements, we obtained the EDS spectra of Pd -based samples, an example of which for Au-NRs/sputtered Pd is shown in Figure S3 (Supplementary Materials). It can be seen that no Pt deposition on the WE can be detected. 

**Figure 8:**
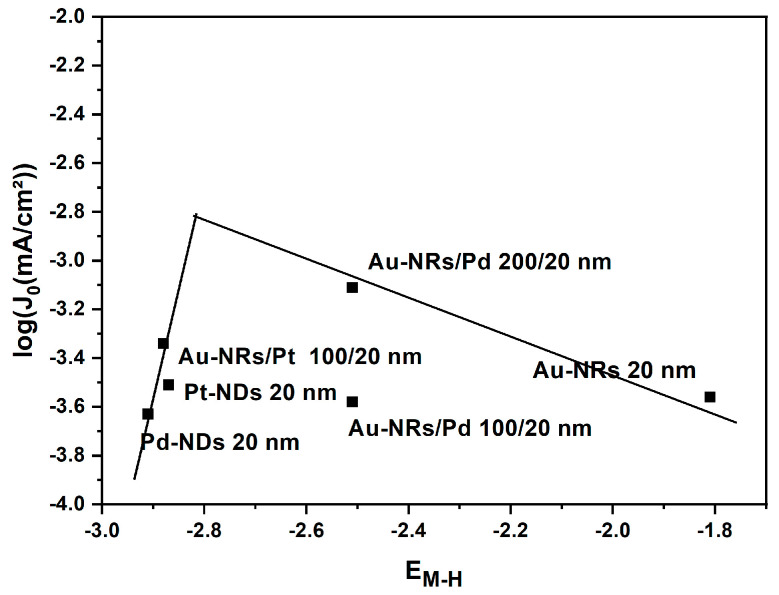
Exchange current density, log(J_0_), as a function of the calculated hydrogen adsorption energies. Only the stable adsorption sites were considered for each structure. Au-NRs/Pd is located on the top of the volcano; the Au-NRs/Pt lie below Pt-NDs, indicating a change in the HER mechanism. For the sake of direct comparison, similar nanostructures are considered, e.g., Au-NRs/Pt 100/20 nm and Au-NRs/Pd 100/20.

Figure 8 shows a tentative volcano plot of the hydrogen evolution reaction that relates the exchange current density to the calculated adsorption energies. As mentioned above, Pt is known to be located at the top of the volcano with the highest activity and a moderate adsorption energy, which is strong enough to activate the HER and prevent the formation of stable intermediates that suppress the HER. The mechanisms of HER for Pt and Pd are reported to obey the Volmer–Tafel mechanisms [38,39], although this has been questioned by Durst et al. [23]. Regarding our results and taking into account the Tafel slopes obtained as well as the calculated adsorption energies of H on Pt, Pd, AuPd, and AuPt, there is a trend towards a parallel Volmer–Heyrovsky mechanism with a fast Volmer step. The Au effect on Pd indicates that electronic interactions at the interface between Au and Pd lead to a suitable change in the adsorption energy of H on all Pd sites, and this translates in a fast Volmer–Heyrovsky mechanism. It can be further demonstrated that the interfacial interactions between Au and Pd play a paramount role in controlling performance of Au-NR/Pd 200/20 nm, where an increase in the interfacial area between Au and Pd results in higher activity (without change in the HER mechanism). 

## 5. Conclusions

In conclusion, Au NRs, Au/Pd NRs, and Pt and Pd nanodiscs of 100, 100/20, and 20 nm, respectively, were processed using electrodeposition in AAO template films on Si. Subsequently, the 100 nm Au NRs were used as supports to sputter 20 nm thick Pt and Pd thin layers. Microscopic analysis shows that the sputtering of Pt and Pd layers leads to nanoparticle formation over the entire surface of the nanorods, resulting in a high interfacial area between Au and the sputtered metals. The nanostructures were evaluated as electrocatalysts for the hydrogen evolution reaction via linear sweep voltammetry and Tafel plots. While the electrodeposited nanostructures all show rather moderate performance, the bimetallic Au-NR/sputtered Pt and Pd are characterized by higher activity. Interestingly and most surprisingly, the sputtered Pd on Au NR support outperforms sputtered Pt (on Au-NR). This unusual result (Pt is known to be best catalyst for HER under acidic conditions) is thought to be a direct consequence of the specific electronic interactions between Pd and Au that lead to more suitable hydrogen adsorption energy on the different Pd sites and, consequently, a faster HER mechanism. That the Au-Pd electronic interactions at the Au-Pd interface are beneficial to HER is demonstrated using sputtered Pd on 200 nm Au-NR, which leads to a doubling of the current density (at −0.1 V) in comparison to the shorter NRs. Based on the Tafel slopes, the Heyrovsky–Volmer mechanism is suggested as the dominant mechanism. The results are corroborated by DFT calculations that show that interfacial Pt/Pd–Au electronic interactions are accompanied with charge transfer and redistribution in the d-band of Au, Pt, and Pd. This translates to a d-band shift, which in turn directly affects the hydrogen adsorption energy. In particular, in this study, it is shown that hydrogen adsorbs on all Pd-sites with marginal difference in the adsorption energy, thus leading to better surface coverage and, most importantly, barrier-free surface diffusion and recombination. Finally, based on the results obtained, a tentative volcano plot is proposed.

## Figures and Tables

**Table 1:** Catalyst loading, current density, Tafel slopes, and exchange current density of different structures.

	Catalyst Loading (mg/cm^2^)	Current Density at −0.1 V (mA/cm^2^)	Current Density at −0.1 V (mA/(mg·cm^−2^))	Tafel Slope (mV/dec)	Exchange Current Density log(J_0_) (mA/cm^2^)
Pt-layer 20 nm (Sputtered)	0.063	−3.88	−107.6	57	−3.61
Au-NRs 100 nm	0.044	−1.24	−37.3	139	−3.56
Pt-NDs 20 nm	0.041	−3.69	−109.9	53	−3.51
Pd-NDs 20 nm	0.031	−2.19	−95.6	96	−3.63
Au/Pd NRs 100/20 nm	0.029	−1.58	−55.69	94	−3.80
Au-NRs/Pt (Sputtered) 100/20 nm	0.022	−22.12	−1249.4	24	−3.34
Au-NRs/Pd (Sputtered) 100/20 nm	0.013	−11.36	−1205.3	37	−3.58
Au-NRs/Pd (Sputtered) 200/20 nm	0.008	−40.21	−9327.2	26	−3.11

**Table 2:** Adsorption energies (E_ads_) and hydrogen surface distance (d_H-S_) for Au, Pt, and Pd surfaces.

	E_ads_ (eV)/d_H-S_ (A)			
	Top	Bridge	Fcc	Hcp
Au	−1.57/1.60	−1.75/0.99	−1.81/0.81	−1.62/1.08
Pt	−2.76/1.57	−2.85/1.07	−2.87/0.94	−2.84/0.95
Pd	−2.28/1.57	−2.78/1.05	−2.91/0.87	−2.87/0.88

**Table 3:** Adsorption energies (E_ads_) and hydrogen surface distance (d_H-S_) for AuPt and AuPd surfaces. M = Pt, Pd.

	E_ads_ (eV)/d_H-S_ (A)						
	Top		Bridge			Fcc	Hcp
	Au	M	Au-Au	Au-M	M-M	Au2M	2AuM
AuPt	−2.20/1.59	−2.88/1.57	−2.19/1.36	−2.23/1.49	−2.52/1.12	−2.10/1.60	−2.34/1.33
AuPd	−2.37/1.60	−2.51/1.56	−2.29/1.41	−2.25/1.65	−2.48/1.11	−2.28/1.50	−2.29/1.39

## Data Availability

The data presented in this study are available upon request from the corresponding author.

## Supplementary Materials

The following supporting information can be downloaded at: https://www.mdpi.com/article/10.3390/nano13132007/s1, Figure S1: SEM micrographs and EDS spectra for monolithic structures: (a) Au-NRs; (b) Pt-NDs; (c) Pd-NDs.; Figure S2: Measured and fitted Electrochemical impedance spectroscopy (EIS) results for all synthesized nanostructures.; Figure S3: EDS spectrum of Au-NRs/Pd (sputtered) 100/20 nm after the electrochemical measurements.
