# Supplementary material for: Au-Nanorods Supporting Pd and Pt Nanocatalysts for the Hydrogen Evolution Reaction: Pd Is Revealed to Be a Better Catalyst than Pt
Source: Nanomaterials (Basel). 2023 Jul 5;13(13):2007. doi: 10.3390/nano13132007 (PMC10343570; doi:10.3390/nano13132007)
Supplement: Supplementary file 1 [file nanomaterials-13-02007-s001.zip › nanomaterials-2480289-supplementary.pdf]

Supplementary Information

Au-Nanorods Supporting Pd and Pt Nanocatalysts for the Hydrogen Evolution Reaction: Pd Is Revealed to Be a Better Catalyst Than Pt

Ayoub Laghrissi [1] and Mohammed Es-Souni [2,*]

1 Currently with the Technical Faculty, Mads Clausen Institute, University of Southern Denmark, 6400 Sonderborg, Denmark; laghrissi@mci.sdu.dk

2 Formerly with Kiel University of Applied Sciences, Grenzstrasse 3, D-24149 Kiel, Germany

* Correspondence: mohammed.es-souni@fh-kiel.de

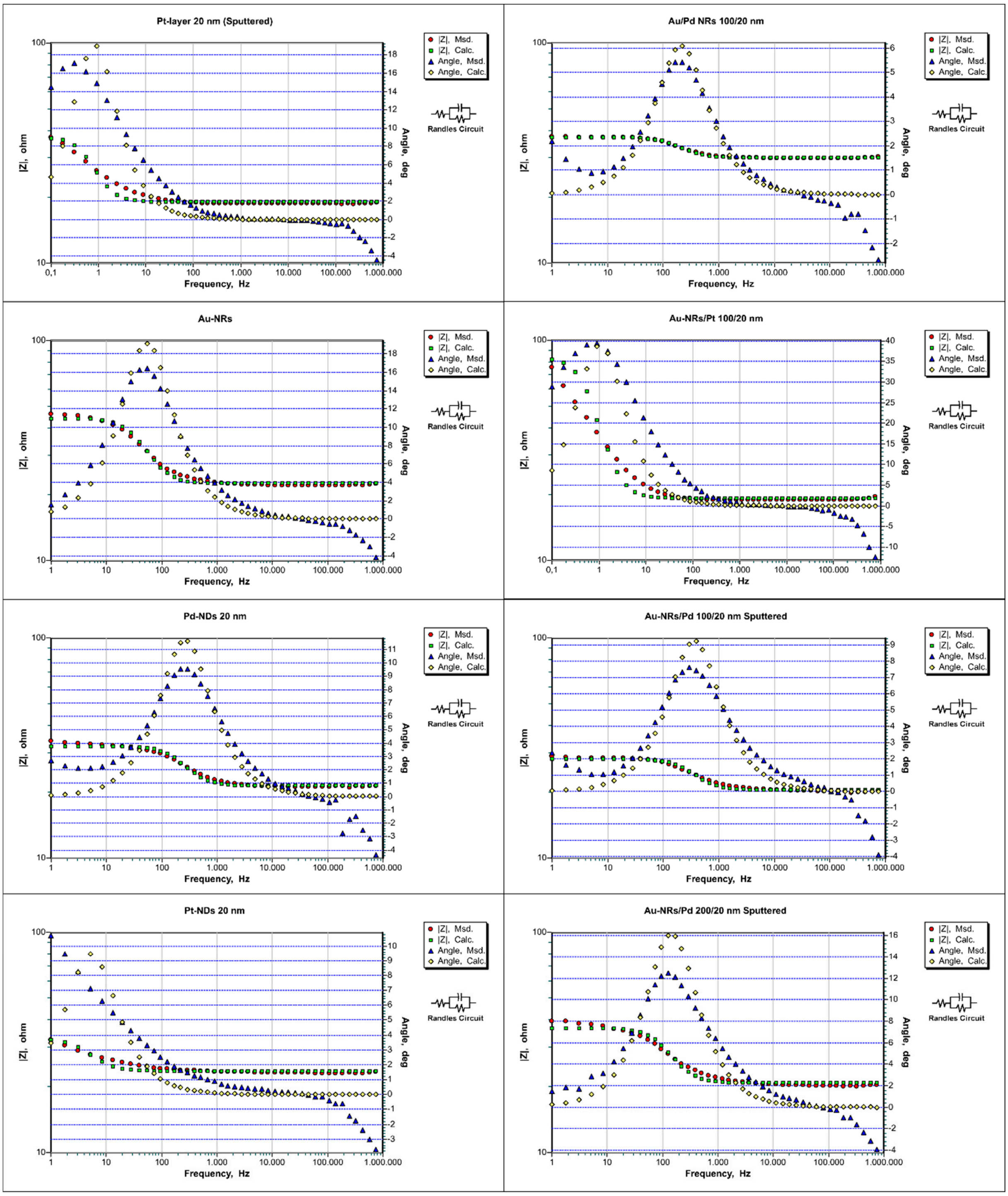

Figure. S1: Measured and fitted Electrochemical impedance spectroscopy (EIS) results for all synthesized nanostructures.

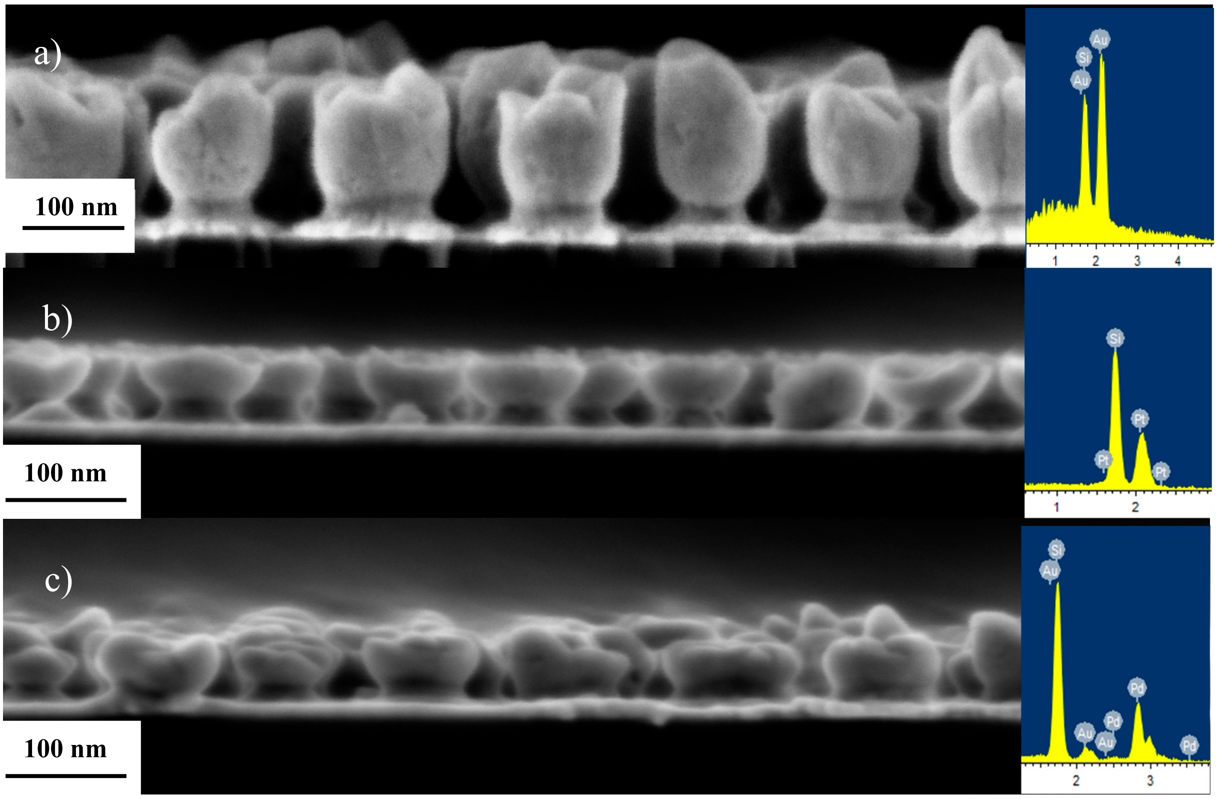

Figure. S2 SEM micrographs and EDS spectra for monolithic structures: **(a)** Au-NRs; **(b)** Pt-NDs; **(c)** Pd-NDs.

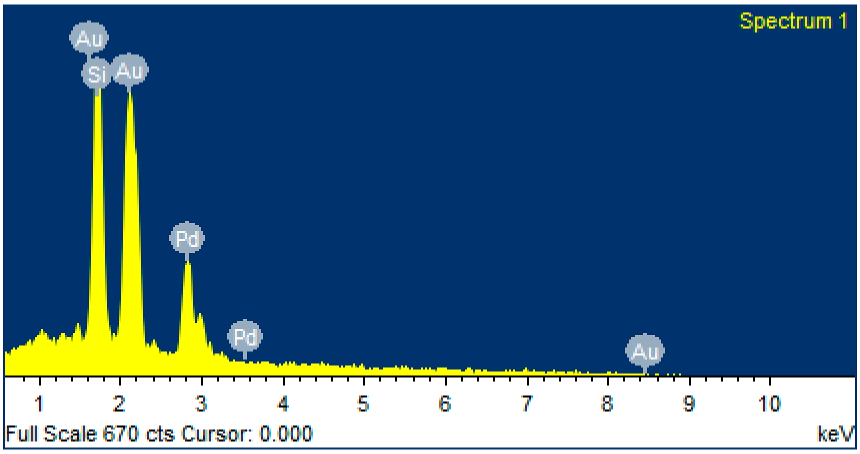

Figure. S3 EDS spectrum of Au-NRs/Pd (sputtered) 100/20 nm after the electrochemical measurements.
